# Predictors of Ureteral Strictures after Retrograde Ureteroscopic Treatment of Impacted Ureteral Stones: A Systematic Literature Review

**DOI:** 10.3390/jcm12103603

**Published:** 2023-05-22

**Authors:** Senol Tonyali, Mehmet Yilmaz, Lazaros Tzelves, Esteban Emiliani, Vincent De Coninck, Etienne Xavier Keller, Arkadiusz Miernik

**Affiliations:** 1 Department of Urology, Istanbul Faculty of Medicine, Istanbul University, 34452 Istanbul, Turkey; 2 Department of Urology, Faculty of Medicine, Medical Center, University of Freiburg, 79106 Freiburg, Germany; 3 European Association of Urology, Young Academic Urologist Endourology and Urolithiasis Working Party, NL-6803 Arnhem, The Netherlands; 4 Department of Urology/Uro-Oncology, University College of London Hospitals (UCLH), London NW1 2BU, UK; 5 Department of Urology, Fundacio Puigvert, Autonomous University of Barcelona, 08193 Barcelona, Spain; 6 Department of Urology, AZ Klina, 2930 Brasschaat, Belgium; 7 Department of Urology, University Hospital Zurich, University of Zurich, 8006 Zurich, Switzerland

**Keywords:** stricture, ureteroscopy, impacted stone, ureteral perforation

## Abstract

Background: The stricture-formation rate following ureteroscopy ranges from 0.5 to 5% and might amount to 24% in patients with impacted ureteral stones. The pathogenesis of ureteral stricture formation is not yet fully understood. It is likely that the patient and stone characteristics, as well as intervention factors, play a role in this process. In this systematic review, we aimed to determine the potential factors responsible for ureteral stricture formation in patients having impacted ureteral stones. Methods: Following the Preferred Reporting Items for Systematic Reviews and Meta-Analysis (PRISMA) criteria, we conducted systematic online research through PubMed and Web of Science without a time restriction, applying the keywords “ureteral stone”, “ureteral calculus”, “impacted stone”, “ureteral stenosis”, “ureteroscopic lithotripsy”, “impacted calculus”, and “ureteral strictures” singly or in combination. Results: After eliminating non-eligible studies, we identified five articles on ureteral stricture formation following treatment of impacted ureteral stones. Ureteral perforation and/or mucosal damage appeared as key predictors of ureteral stricture following retrograde ureteroscopy (URS) for impacted ureteral stones. Besides ureteral perforation stone size, embedded stone fragments into the ureter during lithotripsy, failed URS, degree of hydronephrosis, nephrostomy tube or double-J stent (DJS)/ureter catheter insertion were also suggested factors leading to ureteral strictures. Conclusion: Ureteral perforation during surgery might be considered the main risk factor for ureteral stricture formation following retrograde ureteroscopic stone removal for impacted ureteral stones.

## 1. Introduction

Urolithiasis is a major health problem worldwide, with an estimated incidence ranging from 5% to 12% in developed countries. Since the refinement of endoscopic devices, ureteroscopic stone surgery has become the most common approach to treating urinary stones [1]. Although considered an effective and minimally invasive procedure, ureteroscopy remains associated with particular complications such as mucosal abrasion, false passage, ureteral perforation, ureteral avulsion, thermal injury, and intussusception. Some of the late postoperative complications are vesicoureteral reflux, stricture, and ureteral necrosis [2]. Extracorporeal shock wave lithotripsy (SWL) might also be an alternative option for treatment for ureteral stones; however, it has been shown that ureteroscopic lithotripsy has higher stone-free rates and lower re-treatment rates compared to SWL [3].

Ureteral stricture is lumen narrowing resulting in clinically relevant obstruction. It can develop as a consequence of ureteral instrumentation during stone extraction. The rate of stricture formation following ureteroscopy (URS) varies from 0.5 to 5% and can rise to 24% in patients with impacted ureteral stones [4].

The pathogenesis of ureteral stricture formation is not yet fully understood. The patient and stone characteristics, as well as intervention features, likely play a role in this process. There is evidence that ureteral injuries such as perforation, avulsion, and thermal damage can significantly facilitate stricture formation. Other factors such as employing large caliber instruments, stone impaction, and excessive operation times are also potential reasons for ureteral stricture [4,5].

There is no consensus on how to define stone impaction. Some literature sources describe it as follows: 1. the inability to pass a guidewire beyond the ureteral stone during the first attempt; 2. mild to severe hydronephrosis caused by the stone; 3. the stone remaining at the same location for 2 months [6].

There is a lack of robust data in the literature regarding impacted ureteral stones and their effect on ureteral stricture occurrence after endoscopic treatment. In this systematic review, we aimed to determine the factors potentially responsible for ureteral stricture formation in patients having impacted ureteral stones.

## 2. Material and Methods

**Figure 1:**
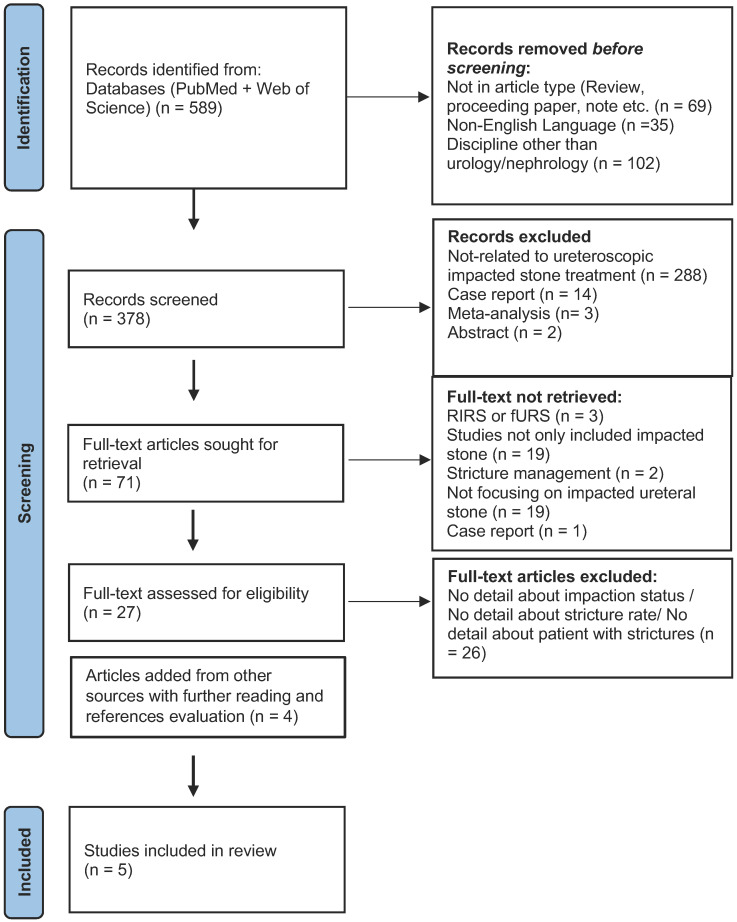
Identification of studies via databases and registers. (Abbreviation: Retrograde intrarenal surgery-RIRS).

Relying on the PRISMA criteria [7,8], we conducted online systematic research through PubMed and Web of Science without a time restriction, utilizing the keywords “ureteral stone”, “ureteral calculus”, “impacted stone”, “ureteral stenosis”, “ureteroscopic lithotripsy”, “impacted calculus”, and “ureteral strictures” singly or in combination. References in all included studies were also scanned to identify additional information sources. The language of eligible studies was restricted to English (Figure 1). The study protocol was not registered to PROSPERO since it is a prospective register of systematic reviews.

### 2.1. Inclusion and Exclusion Criteria

The studies included were selected according to the following criteria: (1) clinical studies addressing ureteric strictures following ureterorenoscopic treatment of impacted ureteral stones; (2) outcomes describing at least one of the following in addition to the presence of post-ureteroscopic ureteric stricture: preoperative patient data [stone size, impaction status, presence of double-J stent (DJS) or nephrostomy tube], and intraoperative complications such as ureteric perforation. Accordingly, studies investigating patients without stone impaction and those who underwent surgeries other than ureteroscopic stone treatment were excluded.

Meanwhile, repeated publications, conference proceedings, non-published materials, editorials, or reviews were also excluded from this analysis. Two reviewers conducted the data source search and extraction independently, and disagreements were resolved by open discussion.

Case reports, case series, review articles, letters to editors, and articles not pertaining to ureteral strictures following impacted ureteral stone treatment were excluded from the study by titles, abstracts, and full-text screening.

### 2.2. Data Extraction

Data were extracted independently by two reviewers using a pre-defined Excel datasheet with the following variables: “first author”, “year of publication”, “general patients’ characteristics”, “stones characteristics (size, location, amount)”, “study design”, “type of surgical technique”, and relevant medical conditions (nephrostomy tube insertion, Double J stent placement) related to ureteral stricture.

### 2.3. Primary Outcomes

The primary outcome in this systematic review was ureteral strictures following ureteroscopy for impacted ureteral stones. Only studies in English were included.

## 3. Results

After eliminating non-eligible studies, we identified five articles addressing ureteral stricture formation following therapy for impacted ureteral stones (Table 1) [6,9,10,11,12]. Our study selection is shown in Figure 1. Because of heterogeneous data and definitions, we conducted no meta-analysis.

Brito et al. [9] reported a 14.2% rate of ureteral stricture in 42 patients having impacted ureteral stones treated via ureteroscopic pneumatic lithotripsy. The rate of strictures in the proximal ureter was higher than those in the mid and distal ureter: 44% versus 8.3% and 4.7%, respectively. In total, four patients with proximal ureter stones developed a ureteral stricture, three of whom also had residual stones.

In their prospective study, Alazaby et al. [11] compared laser lithotripsy alone and in combination with pneumatic lithotripsy in patients with impacted stones in the pelvico-ureteric junction. They reported 2 (4.4%) ureteral strictures in the laser group, whereas none in their combined group.

In their retrospective study, Bayar et al. [10] discovered that ureteral perforation during ureteroscopy for impacted ureteral stones was one of the main causes leading to ureteral strictures. They reported that the ureteral stricture rate was almost 14-fold higher in patients who experienced ureteral perforation during ureteroscopy than in patients who did not, 5.6% vs. 80% [10].

Fam et al. [6] prospectively investigated stricture formation in patients undergoing ureteroscopy for impacted ureteral calculi and reported that 7.8% of 64 patients developed ureteral stricture. They found that intraoperative ureter perforation, damage to the ureter mucosa, residual stone impacted on the ureteral wall, stone size, stone side, and duration of impaction are not risk factors for stricture formation.

In a recent article, Al-Nabulsi et al. [12] reported a 3.3% ureteral stricture rate in 297 patients who underwent ureteroscopic treatment for impacted ureteral stones. They concluded that the degree of hydronephrosis, residual fragments, and intraoperative ureteric injury were significant predictors of ureteral stricture formation.

## 4. Discussion

The management of an impacted ureteral stone deserves particular attention. Not only stone and patient characteristics but also surgical techniques, instruments, and postoperative care seem to play a role in ureteral stricture formation. An impacted stone can cause inflammation, urothelial hypertrophy, and interstitial fibrosis in the affected part of the ureter. These conditions can occur due to ischemia related to prolonged endoluminal pressure, as well as immunological reaction to the stone itself [4]. Moreover, the instruments used during surgery might damage the ureter. Using large caliber endoscopes or ureteral access sheaths can trigger ureteral mucosal damage or perforation. Some authors maintain that pressure-induced ischemia and especially long operating times might be a reason for ureteral stricture [13]. In a retrospective study examining the etiology of ureteral strictures in a single institution, the main predisposing factor for stricture was urolithiasis with a rate of 60%. The complications encountered during endoscopic stone treatment were ureteral perforation, perforation with infected urinoma, fractured guidewire left in situ, and ureteric orifice resection [14].

In our systematic review, we identified ureteral perforation and/or mucosal damage as two of the most important predictors of ureteral stricture following retrograde ureteroscopy for impacted ureteral stones. Besides the ureteral perforation, stone size, stone fragments embedded within the ureter during lithotripsy, failed URS, degree of hydronephrosis, nephrostomy tube, or DJS/ureter catheter insertion are also suggested factors leading to ureteral strictures.

In a big patient cohort including 4875 patients 1.94% (95/4875) of patients developed ureteral stricture following ureteroscopic lithotripsy. Among 19 patients with a known medical history, 11 had impacted ureteral calculi and two were complicated with ureteral perforations [1].

Pneumatic lithotripsy might also be an option for retrograde ureteroscopic impacted stone treatment. Pneumatic lithotripsy was utilized particularly before the wide use of lasers. For more than 10 years, the usage of pneumatic lithotripsy has dramatically decreased; however, it is still a viable option in developing or non-developed countries [15]. It has been shown that the stone-free rate of pneumatic lithotripsy is lower than laser lithotripsy [16,17] on the other hand complication rate is comparable between the two modalities [17]. Interestingly in a study comparing pneumatic lithotripsy and laser lithotripsy not only in impacted but also non-impacted stone, the ureteral stricture rate was significantly higher in the laser lithotripsy group, 1% vs. 4.9%, *p* = 0.02. The other complications such as perforation or bleeding were comparable between the two groups [18]. Ureteral stricture following laser and pneumatic lithotripsy might be related to the mechanism of actions of both types of lithotripsy, such as laser lithotripsy utilizing photothermal whereas pneumatic lithotripsy utilizes a jack-hammer effect [19].

Different techniques such as antegrade ureterorenoscopy and laparoscopic ureterolithotomy (LU) may also be utilized in the management of impacted ureteral stones. In a study by Guler et al. [20] comparing retrograde intrarenal surgery, antegrade URS, and LU, the authors reported that antegrade URS or LU are more logical options than RIRS in the management of ureteral stones larger than 1.5 cm. They also reported a ureteral stenosis rate of 4.3% in the RIRS group; however, there was no ureteral stenosis in the other two groups. Moreover, in another study investigating the risk of ureteral stricture formation following retroperitoneal LU and URSL, the authors did not find any significant difference between the ureteral stricture rates of the two groups. The stone-free status of retroperitoneal LU was higher compared to URSL; however, retroperitoneal LU was also associated with more complications. Furthermore, there was no information related to the stone impaction status of this patient cohort [21]. Shao et al. [22] reported that retroperitoneal LU is superior to URSL in terms of stone-clearance rates in patients having impacted ureteral stones larger than 12 mm, 97.1 vs. 89.9%, *p* = 0.017. After a median follow-up of 20 months, 3.6% of patients in the URSL group and 1.5% of patients in the retroperitoneal LU group developed ureteral stricture which was not statistically significant. However, strictures requiring surgical intervention were significantly higher in the URSL group compared to the retroperitoneal LU group, 2.9% vs. 0%, *p* = 0.046 [22]. This increase might be related to the coagulation and ablation effect of the laser as well as extensive intra-ureteral manipulation. Since in LU cold knife is usually utilized and there is no intra-ureteral manipulation.

Although their patient cohort was heterogenous in stone-impaction terms, Elashry et al. [23] reported on their 15 years of experience managing lower ureteric stones in 4512 patients involving 5133 procedures. They detected 58 (1.13%) ureteral perforations in their series, of which 36 were impacted stones. Ureteric strictures developed in 12 (0.23%) patients. All 12 patients presented impacted stones, and six of them experienced significant or insignificant ureteral perforations during surgery. In their study, Morgentaler et al. report on their findings on managing impacted ureteral calculi in 42 patients. Although they did not carry out strict follow-ups after stone removal, they reported that two patients (4.7%) developed a ureteral stricture: one experienced a false passage during stone removal and the other patient’s medical history revealed previous unsuccessful endoscopic stone retrieval [24]. However, their patients had undergone various types of interventions previously such as ESWL or stone basket extraction before laser lithotripsy, making it difficult to come to any conclusions about their results. In another study, Roberts et al. reported a 24% rate of ureteral stricture following stone removal in patients with impacted stones. They concluded that a ureteral perforation on the side of stone impaction is the primary risk factor [4]. However, their study included retrograde ureteroscopy and percutaneous antegrade ureteroscopy, as well as laparoscopic ureterolithotomy and open ureterolithotomy.

Stone side, size, and location have also been suggested as risk factors for stricture. In a patient cohort of May et al. [25], the ureteral stricture rate following ureteroscopy for ureter stones was 2 times higher in the left ureter compared to the right ureter. The authors suggested that the left ureter might have an anatomic or functional predisposition to obstruction. However, this suggestion has not been widely supported by other studies.

The aforementioned reasons lead us to mention some preventive measures that might help lower the likelihood of ureteral stricture formation. First of all, preserving kidney function while removing the stone must be the primary goal of clinical management. This can be understood as primary and secondary URS. With regard to primary endoscopic treatment, the urologist must consider whether treating a large ureteral calculus during a still emergent event (and accounting for increased peristalsis) may not risk raising the complication rate. Therefore, in certain situations, it is advisable to first enable urine drainage. This can be accomplished by a DJS or nephrostomy tube insertion. The planned therapy can then do secondarily. However, it must be considered that DJS insertion might not be as easy as thought. Impaction of the stone to the ureteral wall and pathological changes in the ureter due to impaction may prevent either the guide wire or DJS from passing beyond the stone. This intervention can also result in perforation of the ureter. Therefore, nephrostomy tube placement may be a more rational option in patients with impacted stones. Since prolonged stone impaction exacerbates pathological anomalies, definitive intervention should be performed at the earliest convenience. A feeling of being on the safe side by inserting DJS or a nephrostomy tube might cause a ureteral stricture over the long term. Secondly, the type of surgery is another important topic. As a ureter with an impacted stone is potentially vulnerable, extra care must be taken taking to avoid mucosal and ureteral damage. If it is difficult to access the impacted stone via retrograde ureteroscopy, anterograde ureteroscopy, or laparoscopic/open ureterolithotomy could be boldly considered. Hu et al. [21] also compared stricture formation rates between patients who underwent retroperitoneal laparoscopic ureterolithotomy (RPLU) and ureteroscopy with holmium laser (URSL), reporting that stricture formation was similar in RPLU and URSL groups, 2% vs. 2.5%, respectively, *p* = 1.000. After adjusting for confounding factors, neither RPLU nor URSL proved to be significantly related to postoperative stricture formation. However, initial and first-month stone-free rates were significantly higher in the RPLU group than the URSL group, 100% vs. 78.6% and 100% vs. 82.4%, respectively, *p* = 0.000 for both. This might suggest that surgical features of ureteroscopic lithotripsy such as the instruments’ caliber, mucosal damage, or ureteral ischemia might contribute significantly to stricture formation. Although there is no definitive threshold for stone size favoring LU or RPLU, 12, 15 mm or 20 mm might be determined to select the laparoscopic approach. In addition, it may be difficult to perform repeated LU and RPLU as there is a high risk of postoperative adhesions.

The principle of stricture management might be early diagnosis, symptom relief, and preservation of renal function. Endourological interventions as well as re-implantation and reconstruction surgeries or nephrectomy as a last resort might be utilized in patient management. Reconstructive surgery has been shown to be significantly superior to endourological procedures in terms of success rate [1]. In a study by May et al., the success rate of endourological management of ureteral stricture was 27.5% [25]. However, endourological procedures might be the treatment of choice in short ureteral strictures (particularly shorter than 1 cm) since those are remarkably superior to reconstructive surgeries in terms of operative time, intraoperative blood loss, and surgical morbidity. Faster recovery time for endourological procedures is also another issue that makes them widely preferred [1].

Despite being a highly invasive treatment, open ureterolithotomy and resecting the pathological ureter via ureteroureterostomy were suggested in patients with impacted ureteral stones inaccessible via a ureteroscope. Xi et al. [26] compared stone-free and ureteral strictures rates in patients presenting impacted ureteral stones who underwent ureterorenoscopic lithotripsy (URSL) (n = 61) and open ureterolithotomy, partial resection of affected ureter and ureteroureterostomy (OUU) (n = 25). They reported a higher rate of ureteral stricture in their URSL group than the OUU group, 26.2% vs. 4%, *p* = 0.019.

We think another issue warranting discussion regarding stricture formation is the lack of urine passing through the ureter. O’Sullivan et al. investigated the rate of ureteral strictures in patients who had undergone ureteric instrumentation for some reason while having a nephrostomy tube in place. Their study included 18 patients. A nephrostomy tube was left on free drainage after instrumentation in eleven patients and a double J stent was inserted and/or nephrostomy was closed in seven patients. Overall, eight of eleven patients developed ureteral strictures, but none of the remaining seven patients did. The authors concluded that a traumatized ureter must not be allowed to remain dry and unstented [27].

Due to the paucity of well-conducted studies, it is not possible to specify the risk factors contributing to ureteral stricture formation following ureteroscopy for impacted stones. Ureteral perforation during surgery seems to be the main risk factor for ureteral stricture formation following retrograde ureteroscopic stone removal for impacted ureteral stones. The patients and their stone characteristics, the duration of impaction, and surgical factors operative might play a role in stricture formation.

### Limitations

The main limitation of this study is the relatively low number of studies included. We identified several manuscripts in the literature reporting ureteral strictures following ureteroscopic treatment of ureteral stones, but they are very methodologically heterogeneous. Our main aim in this study was to clarify the factors leading to ureteric stricture formation in patients with impacted ureteral stones. However, the literature dedicated to this subject is too sparse and the information is not specific enough about patients and procedural characteristics.

## 5. Conclusions

Impacted ureteral stones and their management might be frustrating for urologists as postoperative complications such as strictures can occur. Both preoperative and intraoperative factors can lead to ureteral strictures following ureteroscopic stone removal. Ureteral perforation during surgery can be considered the main risk factor for ureteral stricture formation following retrograde ureteroscopic stone removal for impacted ureteral stones.

## Figures and Tables

**Table 1:** Summary of the studies in the literature on ureteroscopic lithotripsy for impacted ureteral stones.

Author and Year	Study Design	Patient/Procedure Number	Mean Patient Age	Stone Size	Stone Location			Treatment	Stricture Rate	Suggested Risk Factors	Follow-Up
					Proximal Ureter	Mid Ureter	Distal Ureter				
Fam et al., 2015 [6]	Prospective	64		Up to 20 mm	21 (32.8%)	15 (23.4%)	28 (43.7%)	Retrograde Ureteroscopic laser lithotripsy	5 (7.8%)	None among intraoperative or stone-related factors	6 months
Al-Nabulsi et al., 2021 [9]	Retrospective	297	52 (20–86)					Retrograde Ureteroscopic laser lithotripsy	10 (3.3%)	Stone size, degree of hydronephrosis, Nephrostomy insertion, mucosal injury, ureteric perforation, residual stone fragments, embedded stone fragments *	3 months
Stricture Group		10	58	11.5	5 (50%)	3 (30%)	2 (20%)				
Without stricture Group		287	53	7.6	119 (41.5%)	43 (15%)	125 (43.5%)				
Alazaby et al., 2020 [10]	Prospective comparative	90						Laser alone, combined (laser + pneumatic)	2 (2.2%)	Ureter mucosa laceration and embedded stone particles	Na
Laser Group		45	51 ± 9.7	12.8 ± 1.1					2 (4.4%)		
Combined Group (Laser + Pneumatic)		45	49 ± 11.4	13.1 ± 1.4					0		
Brito et al., 2006 [11]	Retrospective	42	23–72	5–20 mm	9	11	22	Retrograde Ureteroscopic pneumatic lithotripsy	14.2%	Ureteral perforation (treatment of proximal ureteric impacted stone has high risk of perforation)	Na
Bayar et al., 2016 [12]	Retrospective	81	39 ± 16	11.5 ± 5.2	31 (38%)	16 (20%)	34 (42%)	Retrograde Ureteroscopic stone removal (laser/pneumatic)	5.6% (4/71 in non-perforated) 80% in perforated	Perforation	3–86 months

* Significantly higher in the stricture group. URSL: ureteroscopic lithotripsy. OUU: open ureterolithotomy, resection of concurrent pathologic ureter, and ureteroureterostomy. ESWL: extracorporeal shockwave lithotripsy.

## Data Availability

Not applicable.
